# E2F1 promotes cell cycle progression by stabilizing spindle fiber in colorectal cancer cells

**DOI:** 10.1186/s11658-022-00392-y

**Published:** 2022-10-11

**Authors:** Zejun Fang, Min Lin, Shenghui Chen, Hong Liu, Minjing Zhu, Yanyan Hu, Shanshan Han, Yizhang Wang, Long Sun, Fengjiao Zhu, Chengfu Xu, Chaoju Gong

**Affiliations:** 1 Central Laboratory, Sanmen People’s Hospital of Zhejiang Province, Sanmen, 317100 China; 2 Department of Clinical Laboratory, Sanmen People’s Hospital of Zhejiang Province, No. 15 Taihe Road, Hairun Street, Sanmen, 317100 China; 3 Department of Gastroenterology, The First Affiliated Hospital, Zhejiang University School of Medicine, No. 79 Qingchun Road, Hangzhou, 310003 China; 4 Department of Gastroenterology, Affiliated Hangzhou First People’s Hospital, Zhejiang University School of Medicine, Hangzhou, 310000 China; 5 Department of Microbiology, Immunology and Inflammation, Lewis Katz School of Medicine, Temple University, Philadelphia, PA 19140 USA; 6 Department of Gastrointestinal Surgery, Sanmen People’s Hospital of Zhejiang Province, Sanmen, 317100 China; 7 Central Laboratory, The Affiliated Xuzhou Municipal Hospital of Xuzhou Medical University, No. 19 Zhongshan Bei Road, Xuzhou, 221100 China

**Keywords:** E2F1, Stathmin1, TACC3, Colorectal carcinoma, Spindle fiber, Cell cycle

## Abstract

**Background:**

E2F1 is a transcription factor that regulates cell cycle progression. It is highly expressed in most cancer cells and activates transcription of cell cycle-related kinases. Stathmin1 and transforming acidic coiled-coil-containing protein 3 (TACC3) are factors that enhance the stability of spindle fiber.

**Methods:**

The E2F1-mediated transcription of transforming acidic coiled-coil-containing protein 3 (TACC3) and stathmin1 was examined using the Cancer Genome Atlas (TCGA) analysis, quantitative polymerase chain reaction (qPCR), immunoblotting, chromatin immunoprecipitation (ChIP), and luciferase reporter. Protein–protein interaction was studied using co-IP. The spindle structure was shown by immunofluorescence. Phenotype experiments were performed through MTS assay, flow cytometry, and tumor xenografts. Clinical colorectal cancer (CRC) specimens were analyzed based on immunohistochemistry.

**Results:**

The present study showed that E2F1 expression correlates positively with the expression levels of stathmin1 and TACC3 in colorectal cancer (CRC) tissues, and that E2F1 transactivates stathmin1 and TACC3 in CRC cells. Furthermore, protein kinase A (PKA)-mediated phosphorylation of stathmin1 at Ser16 is essential to the phosphorylation of TACC3 at Ser558, facilitating the assembly of TACC3/clathrin/α-tubulin complexes during spindle formation. Overexpression of Ser16-mutated stathmin1, as well as knockdown of stathmin1 or TACC3, lead to ectopic spindle poles including disorganized and multipolar spindles. Overexpression of wild-type but not Ser16-mutated stathmin1 promotes cell proliferation in vitro and tumor growth in vivo. Consistently, a high level of E2F1, stathmin1, or TACC3 not only associates with tumor size, lymph node metastasis, TNM stage, and distant metastasis, but predicts poor survival in CRC patients.

**Conclusions:**

E2F1 drives the cell cycle of CRC by promoting spindle assembly, in which E2F1-induced stathmin1 and TACC3 enhance the stability of spindle fiber.

**Supplementary Information:**

The online version contains supplementary material available at 10.1186/s11658-022-00392-y.

## Introduction

Colorectal carcinoma (CRC) is the third most common cancer, and the second leading cause of cancer-related deaths worldwide with nearly 0.9 million deaths each year, accounting for approximately 10% of all confirmed cancer cases and cancer-related deaths [1, 2]. Because of increased use of sigmoidoscopy and colonoscopy with polypectomy for early diagnosis and treatment, the prognosis of patients with CRC has improved to some extent in recent years [3]. Regrettably, most established therapies including surgical resection, chemotherapy, radiotherapy, and immunotherapy are not fully effective, especially for those CRC patients with late metastatic stage, whose prognoses remain poor [4].

E2F transcription factors (E2Fs) can activate or silence many oncogenes or tumor suppressor genes in various malignancies, which have been shown to be involved in cellular proliferation, differentiation, apoptosis, metastasis, and chemoresistance in colorectal cancer [5, 6]. As the first member discovered, E2F1 is a transcription co-factor that interacts with retinoblastoma protein (RB) to regulate the transcription of cell cycle-related proteins [6]. The cyclin dependent kinase 4 (CDK4)-pRB-E2F1 axis has been shown to be essential for cell cycle progression in normal and cancer cells, and high expression of E2F1 has been found to promote the proliferation of cancer cells by transactivating cell cycle-related kinases [7, 8]. Moreover, because E2F1 is a critical transcription factor, it may also modulate cell cycle progression through its regulation of the transcription of other genes [5].

Stathmin1 and transforming acidic coiled-coil-containing protein 3 (TACC3) are two microtubule binding factors that are highly expressed in cancer cells and that maintain the stability of microtubules [9, 10]. Stathmin1 is involved in the promotion of tubulin filament depolymerization [11]. Phosphorylation of stathmin1 at Ser16 increases during G2/M-phase and stabilizes microtubules during the regulation of mitotic spindles [12]. TACC3 is a clathrin binding protein [13]. Phosphorylation of TACC3 at Ser558 facilitates its interactions with clathrin and tubulin, thus maintaining the stability of spindle fiber arrays [14, 15]. Theoretically, stathmin1 and TACC3 function similarly, but no study to date has reported interactions between these two factors.

The present study evaluated the interactions of E2F1 with stathmin1 and TACC3 in pathological tissues from patients with CRC. E2F1 expression was found to correlate positively with stathmin1 and TACC3 expression in tumor tissues from CRC patients. E2F1 was found to transactivate stathmin1 and TACC3 in CRC cells. Furthermore, phosphorylation of stathmin1 at Ser16 was essential for the phosphorylation of TACC3 at Ser558, facilitating the formation of TACC/clathrin/α-tubulin complexes and maintaining the stability of spindle fibers. These findings indicate that E2F1 not only promotes cell cycle progression through the cyclin-CDK axis, but also modulates cell cycle progression by stabilizing spindle fibers in CRC cells.

## Materials and methods

### Reagents and antibodies

McCoy’s 5A modified medium (Mc5AM), Ham’s F-12 K (Kaighn’s) medium (F-12 K), Leibovitz’s L-15 medium (L-15), fetal bovine serum (FBS), Lipofectamine 3000, and Lipofectamine RNAi Max were purchased from Thermo Fisher (Carlsbad, CA). RPMI-1640 medium (1640) and Dulbecco’s modified Eagle’s medium (DMEM) were obtained from GE Healthcare Inc. (Lafayette, CO). Antibodies against E2F1 (3742), stathmin1 (13655S), p-stathmin1 (Ser16) (4191S), TACC3 (8069S), p-TACC3 (Ser558) (8842S), clathrin (4796), Ki-67 (9449), and normal rabbit IgG (2729) were purchased from Cell Signaling Technology (Danvers, MA). Antibodies against β-actin (E021020) and α-tubulin (E021030), and FLAG-tagged and HRP-conjugated secondary antibodies against rabbit IgG (E022230-01; E030120) and mouse IgG (E022060-01; E030110) were obtained from EarthOx (San Francisco, CA). 100 μM bucladesine (Selleck, Texas, USA, S7858) was used to activate PKA.

### Cell culture

The CRC cell lines HT29, HCT8, DLD1, RKO, SW480, SW620, and HTC116 were purchased from the American Type Culture Collection (ATCC, Manassas, VA). HT29 cells were cultured in Mc5AM; HCT8 and HCT116 cells were cultured in 1640; DLD1 cells were cultured in DMEM; RKO cells were cultured in F-12 K; and SW480 and SW620 cells were cultured in L-15. Each culture medium was supplemented with 10% fetal bovine serum (FBS) and all cells were cultured at 37 °C in an atmosphere containing 5% CO_2_. The identities of all cell lines were authenticated by STR profiling, performed by Cobioer Bioscience Co., Ltd. (Nanjing, China). All experiments were performed within < 10 passages after authentication.

### RNA isolation and qPCR

Total RNA was isolated with RNAiso Plus (TaKaRa, Kyoto, Japan). RNA quality and concentration was evaluated using a Nanodrop ND-1000 Spectrophotometer. A total of 0.5 μg RNA was reverse transcribed using cDNA Reverse Transcription Kit (TaKaRa) according to the manufacturer’s instructions. The acquired cDNA was analyzed in triplicate by real-time PCR on a Life Technologies Step One Plus Real-Time PCR System with SYBR Green Master Mix (Roche, Basel, Switzerland). The results of three independent experiments are presented as mean ± standard deviation.

### Analyses of clinical CRC samples

To analyze the correlations among E2F1, stathmin1, TACC3, and Ki-67 expression, pathological tissue samples (*n* = 231) freshly obtained from CRC patients undergoing surgery at Sanmen People’s Hospital (Taizhou, China) were fixed in 4% formalin, processed routinely, and embedded in paraffin. Sections of 4 μm were placed on glass slides (Thermo Fisher) and immunohistochemically analyzed using primary antibodies against E2F1, stathmin1, p-stathmin1, TACC3, p-TACC3, and Ki-67, as described previously [16]. To assess the score for each slide, eight 200 × fields were selected and 100 cancer cells counted in each field. Immunostaining intensity was divided into four grades: 0, negative; 1, weak; 2, gentle; and 3, strong. The proportions of positively stained cells were classified into five grades: 0, < 5%; 1, 6–25%; 2, 26–50%; 3, 51–75%; and 4, > 75%. Staining results were evaluated and confirmed by two independent investigators blinded to the clinical data. The positive rate of tumor cells and staining intensity were multiplied to acquire immunohistochemistry (IHC) scores. Scores 0–6 were defined as low expression and 7–12 as high expression. Cases with inconsistent scores were further discussed to reach a consensus. Differentiation stages of the tissue samples were assessed at the Sanmen People’s Hospital of Zhejiang Province. The study protocol was approved by the Ethics Committee of Sanmen People’s Hospital of Zhejiang Province, and all patients provided written informed consent.

### Immunoblotting analysis

Whole proteins were extracted from cells by incubation in RIPA lysis buffer (Merck-Millipore, Billerica, MA) for 30 min on ice. The samples were centrifuged at 12,000 ×*g* for 15 min to remove debris, and an aliquot of each supernatant was boiled in SDS-PAGE loading buffer for 5 min. The samples were electrophoresed on SDS-PAGE gels and transferred to nitrocellulose membranes as described previously [16]. Target bands were quantified using Image J software by normalizing to an internal reference. The results of three independent experiments are presented as mean ± standard deviation.

### Vectors and siRNAs

E2F1, stathmin1, TACC3 stathmin-FLAG, and stathmin-MS16-Ala-FLAG (S16 ATA to GGC) expression vectors were purchased from Oligobio (Beijing, China). Each of these sequences was cloned into pCMV or pCMV-c-FLAG vectors. E2F1, stathmin1 and TACC3-specific siRNAs (Additional file 1: Table S1) were purchased from Genepharma (Shanghai, China). The promoters of *STMN1* [nucleotides (nt) −200 to +266] and *TACC3* (nt −300 to +106) were cloned into pGL4.19, and binding site mutations were introduced into these vectors as described in Fig. 3D. Transfection of plasmids (final concentration: 1.5 μg/ml) and small interfering RNAs (siRNA, final concentration: 30 nM) was conducted using Lipofectamine 3000 (Invitrogen; Thermo Fisher Scientific, Inc.) according to the manufacturer’s instructions.

### MTS proliferation assay

DLD1 and HCT116 cells were seeded onto 96-well plates and transfected with siRNAs or vectors. After transfection, cells were cultured with 100 μl complete medium for 24, 48, and 72 h. A 100 μl aliquot of a 1:9 solution of MTS solution (Promega Biosciences, San Luis Obispo, CA) in DMEM was added to each well, and the cells were incubated for 4 h at 37 °C. The absorbance of each well at 490 nm was measured using a SpectraMax M3 multimode microplate reader (Molecular Devices, Sunnyvale, CA). The results of three independent experiments are presented as mean ± standard deviation.

### Chromatin immunoprecipitation assays

Chromatin immunoprecipitation (ChIP) assays were performed using a SimpleChIP Enzymatic Chromatin IP Kit (Cell Signaling Technology), according to the manufacturer’s instructions. E2F1 binding DNA fragments were co-immunoprecipitated using a specific antibody against E2F1. Immunoprecipitated DNA and input DNA fragments were utilized as templates for chromatin immunoprecipitation and PCR (ChIP-PCR) analysis using PrimeSTAR GXL polymerase mix (TaKaRa, Shiga, Japan). Positive binding sites identified by ChIP-PCR analysis were verified by ChIP-qPCR analysis, performed using TB Green Premix Ex Taq (TaKaRa) and detected using a StepOne Plus System (Applied Biosystems Inc., Carlsbad, CA). The primers used in the ChIP-PCR and ChIP-qPCR analysis are listed in Additional file 1: Table S2. The results of three independent experiments are presented as mean ± standard deviation.

### Luciferase reporter assays

DLD1 cells were seeded onto 24-well plates and transfected with pCMV-E2F1 or pCMV vector, along with pGL4.19-STMN/TACC3-promoter, binding site mutants, and pRL-TK vector for 24 h. The cells were harvested and lysed with 1× passive lysis buffer (Promega Biosciences) at room temperature for 15 min. Luciferase activity was analyzed using the Dual Luciferase Reporter Assay System Kit (Promega), according to the manufacturer’s instructions. Total light production (OD 490 nm) was measured using the SpectraMax M3 multimode microplate reader (Molecular Devices) and normalized relative to light production by renilla. The results of three independent experiments are presented as mean ± standard deviation.

### Cell cycle assessment by flow cytometry

DLD1 or HCT116 cells were mixed with serum-free medium, seeded in 6-well plates (1 × 10^5^ cells/well) and incubated for 24 h to synchronize cell cycle progression. After transfection, the cells were cultured in complete medium for 48 h. Approximately 5 × 10^5^ cells were fixed overnight in 1 ml ethanol, stained with PI/RNase Staining Solution (Cell Signaling Technology) at 37 °C for 30 min, and analyzed on an FC500 flow cytometer (Beckman Coulter, Inc., Brea, CA). The results of three independent experiments are presented as mean ± standard deviation.

### Co-immunoprecipitation

Forty-eight hours after transfection, DLD1cells were resuspended and lysed in 1.5 ml IP lysis buffer (Thermo Fisher) in 10 cm dishes. The samples were centrifuged to remove insoluble debris, 100 μl of each supernatant was removed, and the remainder of each supernatant was divided into three equal aliquots. One aliquot each was incubated with rabbit anti-FLAG (1:400), anti-TACC3 (1:100), and anti-clathrin (1:100) antibodies, followed by incubation with anti-rabbit IgG antibody (1:100). Co-immunoprecipitation was performed using Pierce Protein A/G Magnetic Beads (Thermo Fisher), according to the manufacturer’s instructions. Input and purified proteins were analyzed by immunoblotting.

### Immunofluorescence

DLD1 or HCT116 cells on cover slips were fixed with phosphate-buffered saline (PBS) containing 4% paraformaldehyde, permeabilized with PBS containing 0.2% Triton X-100, blocked with PBS containing 1% BSA, and then incubated overnight at 4 °C with primary antibody against α-tubulin (1:500), as described above. After three washes with PBS, the cover slips were incubated with Alexa Fluor 488-conjugated secondary antibody (4408S, Cell Signaling Technology, Danvers, MA) in the dark for 60 min, followed by three washes with PBS. Nuclei were counterstained with DAPI. Cover slips were mounted and examined under a microscope using the appropriate filters. One hundred fifty cells in each group were used to determine the percentage of the aberrant spindles. The results of three independent experiments are presented as mean ± standard deviation.

### Tumor xenografts

The protocols of all animal experiments were approved by the Animal Experimental Ethics Committee of the First Affiliated Hospital, College of Medicine, Zhejiang University. Briefly, 20 female BALB/c nude mice were randomly divided into three groups of six mice each. Mice were injected subcutaneously with 5 × 10^6^ cells bearing vector, stathmin1-Flag, or M-S16-Flag. When the tumors became measurable, their lengths and widths were measured every 3 days with calipers, and the tumor volumes were calculated. On day 23, the animals were euthanized, and the tumors were excised and weighed.

### Statistical analysis

Continuous variables were presented as mean ± standard deviation, with differences analyzed by two-tailed Student's *t*-tests and analysis of variance (ANOVA), followed by Tukey’s post hoc tests for one-way ANOVA and Sidak post hoc tests for two-way ANOVA. Clinicopathological characteristics were compared by chi-square or Fisher’s exact probability tests, as appropriate. Correlations between variables were analyzed by Pearson’s or Spearman’s rank correlation tests. Overall survival was analyzed by the Kaplan–Meier method and compared by log-rank tests. Cox regression was used to analyze the hazard ratio for overall survival. Statistical analyses were performed using SPSS version 17.0 (IBM Corp., Armonk, NY) and GraphPad Prism version 5.0 software (GraphPad Software Inc., La Jolla, CA), with *P* < 0.05 considered statistically significant.

## Results

### E2F1 is positively correlated with stathmin1 and TACC3 in CRC

**Fig. 1:**
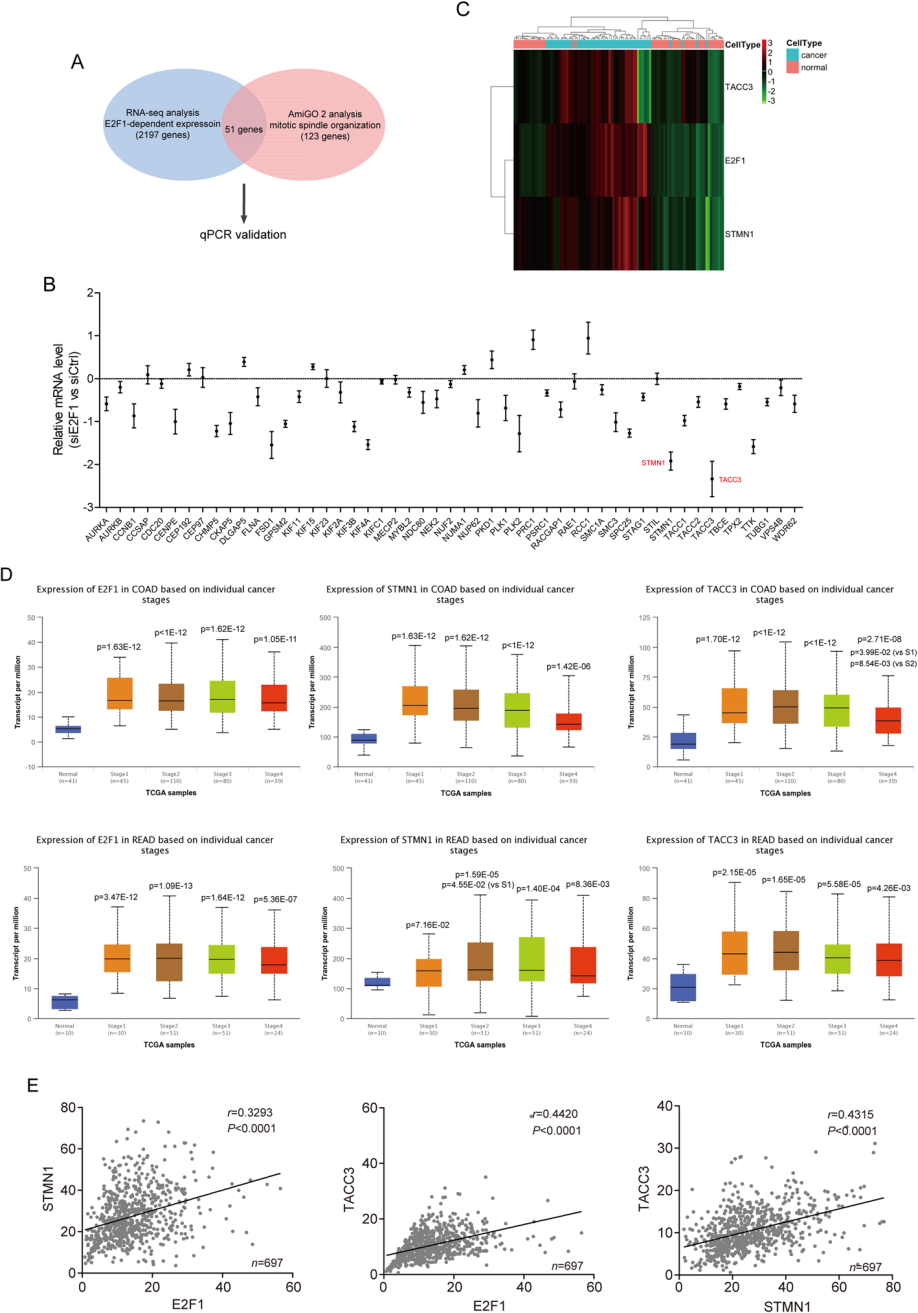
E2F1 is positively correlated with stathmin1 and TACC3 in pathological tissues derived from CRC patients. **A** Differential expression of genes in CRC cells with and without knockdown of E2F1 expression and screening for genes associated with mitotic spindle organization. The 51 screened genes were verified by qPCR. **B** qPCR analysis of relative mRNA levels encoded by these 51 genes in HCT116 cells with and without E2F1 knockdown for 48 h. **C** Differential expression of E2F1, stathmin1, and TACC3 in 104 pairs of CRC tumor and adjacent normal colorectal tissues, based on the TCGA database. Red color represents increased expression and green indicates reduced expression. Expression was normalized using (level_case_-mean_row_)/SD_row_. **D** Effects of cancer stage on levels of expression of E2F1, stathmin1, and TACC3 in 315 COAD and 166 READ cases from the TCGA database and analyzed by UALCAN (http://ualcan.path.uab.edu/analysis.html). The *P*-values (versus normal) were shown without indications and the *P*-values (versus Stage 1 and Stage 2) were indicated as S1 and S2 *COAD* colon adenocarcinoma, *READ* rectum adenocarcinoma **E** Correlations among E2F1, stathmin1, and TACC3 levels in 698 CRC (including COAD and READ) pathological tissue samples from the TCGA database, as determined by linear correlativity. Pearson’s test was used to analyze the correlations

E2F1, a transcription factor highly expressed in cancer cells, has been widely reported to regulate the cell cycle in multiple biological processes, including mitotic spindle organization [17, 18]. To date, however, the roles of E2F1 in mitotic spindle organization have not been clearly determined, especially in CRC. We therefore analyzed the transcriptome profiles of CRC cells with E2F1 knockdown in our previous work [16]. Among the 2197 genes downregulated by E2F1 knockdown, 51 were found to be involved in the gene ontology (GO) item “mitotic spindle organization” (Fig. 1A). Analysis of the levels of expression of mRNAs encoded by these 51 genes in HCT116 cells with and without E2F1 knockdown showed that *STMN1* and *TACC3* were the two genes most significantly downregulated by E2F1 knockdown (Fig. 1B). To determine whether E2F1 expression correlated with the levels of expression of stathmin1 and TACC3 in CRC tissues, their correlation was analyzed using the Cancer Genome Atlas (TCGA) database. This analysis showed that E2F1, stathmin1, and TACC3 were all highly expressed at different pathological stages in tissues derived from CRC patients (Fig. 1C and D), and that the levels of these three factors correlated positively with each other (Fig. 1E).

### Upregulation of E2F1 promotes transactivation of stathmin1 and TACC3

**Fig. 2:**
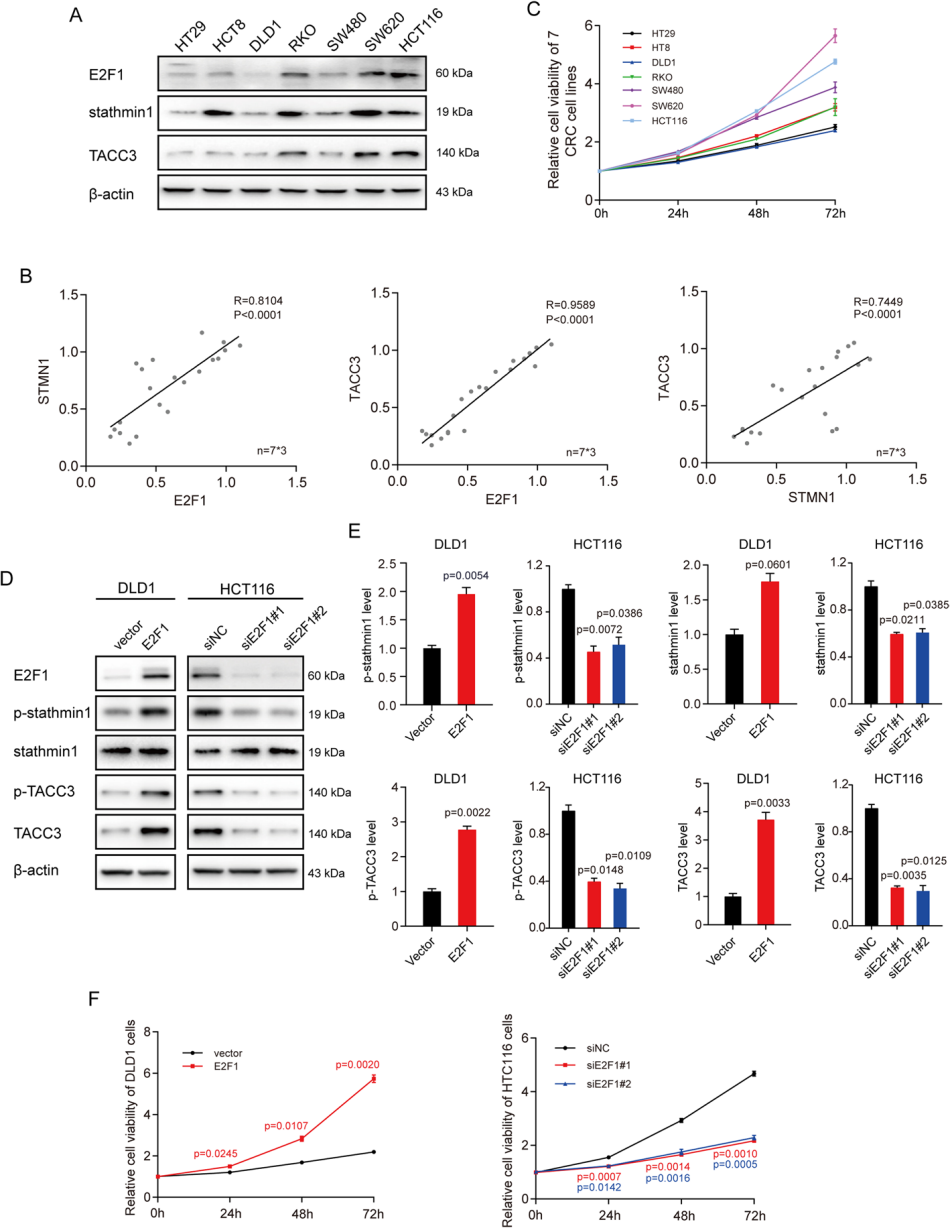
Upregulation of E2F1 promotes the expression of stathmin1 and TACC3 and facilitates cell cycle progression. **A** Total proteins were extracted from HT29, HCT8, DLD1, RKO, SW480, SW620, and HCT116 cells. The levels of E2F1, stathmin1, and TACC3 were determined by immunoblotting. **B** Correlations among E2F1, stathmin1, and TACC3 levels in the CRC cell lines shown in **A**. Pearson’s test was used to analyze the correlations. **C** Viability of the cells shown in **A**, as measured using MTS assays 0, 24, 48, and 72 h after cell seeding. **D** DLD1 cells were transfected with pCMV-E2F1 or pCMV-blank vectors (final vector concentrations, 2 μg/ml). HCT116 cells were transfected with E2F1-specific or control siRNA (final siRNA concentrations, 20 nM). Samples were harvested 48 h after transfection, and the levels of the indicated factors determined by immunoblotting. **E** Relative levels of expression of the proteins indicated in **D**. **F** DLD1 and HTC116 cells were transfected with vectors or siRNAs as described in **D**. Cell viability was measured using MTS assays 0, 24, 48, and 72 h after transfection

Measurements of the expression levels of E2F1, stathmin1, and TACC3 in HT29, HCT8, DLD1, RKO, SW480, SW620, and HCT116 cells showed that these three factors correlated positively with each other in these CRC cell lines (Fig. 2A and B). MTS cell proliferation assay showed that cells with high E2F1 expression grew faster than those with low E2F1 expression (Fig. 2C). The finding that knockdown of E2F1 downregulated the expression of *STMN1* and *TACC3* suggests that E2F1 may positively modulate the expression of stathmin1 and TACC3. This was tested by immunoblotting analysis, which showed that the levels of expression of stathmin1 and TACC3 were increased in E2F1-overexpressing DLD1 cells and were reduced in HCT116 cells transfected with E2F1 targeted siRNAs (Fig. 2D and E). In agreement with previous results [19], we found that E2F1 positively regulated cell cycle progression and cell proliferation (Fig. 2F). A search of the Kyoto Encyclopedia of Genes and Genomes (KEGG) showed no signaling pathway linking stathmin1 and TACC3 to E2F1 in signaling pathways, suggesting that stathmin1 and TACC3 were potential transcriptional products of E2F1.

**Fig. 3:**
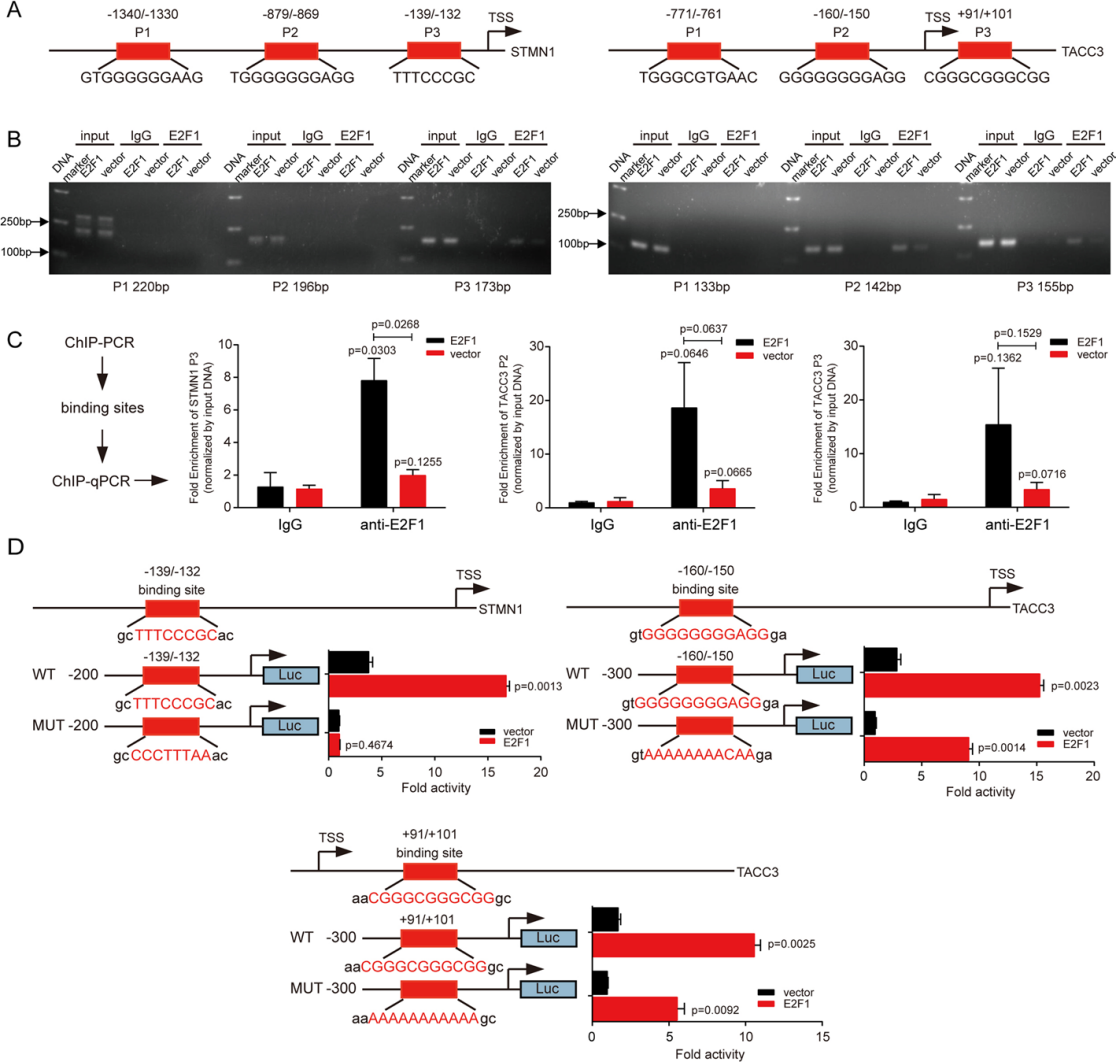
E2F1 transactivates stathmin1 and TACC3 in CRC cells. **A** Schematic representations of the promoter regions of human *STMN1* and *TACC3*. The predicted E2F1 binding sites are indicated. **B** Agarose gel electrophoresis of ChIP-PCR products in the input, IgG, and ChIP groups, showing that E2F1 interacted with the *STMN1* promoter at P3, and with the *TACC3* promoter at P2/P3. **C** Quantitative PCR analysis of the efficiency of interaction of E2F1 DNA at the promoter sites shown in **B**. **D** Luciferase activities of DLD1 cells transfected with pGP4.19 promoters carrying wild-type and mutant binding sites promoter, pRL-TK and vector/E2F1

Analysis of the JSAPAR database identified three potential binding sites for E2F1 on the *STMN1* or *TACC3* promoters. Using ChIP analysis, E2F1-binding DNA fragments on these promoters were co-immunoprecipitated with anti-E2F1 antibody (Fig. 3A). ChIP-PCR and ChIP-qPCR analyses indicated that E2F1 interacted with the *STMN1* promoter at the −139/−132 site and with the *TACC3* promoter at the −160/−150 and +91/+101 sites (Fig. 3B and C). These findings, showing that E2F1 interacts with the *STMN1* and *TACC3* promoters, suggest that E2F1 may transactivate stathmin1 and TACC3.

Using luciferase reporter assays to determine transcriptional activity, we found that the luciferase activity of pGL4.19 vector containing fragments of the *STMN1* promoter was transactivated when DLD1 cells were transfected with pCMV-E2F1 vector. In contrast, little luciferase transactivation was observed when cells were transfected with pGL4.19 vector containing fragments of the −139/−132 mutant *STMN1* promoter. Similarly, the luciferase activity of pGL4.19 vector containing fragments of the −160/−150 or +91/+101 mutant *TACC3* promoter was much lower than that of pGL4.19 vector containing wild type *TACC3* promoter (Fig. 3D). Taken together, these results indicated that E2F1 interacts with the *STMN1* and *TACC3* promoters and promotes the transactivation of stathmin1 and TACC3 in CRC cells.

### Stathmin1 positively regulates the phosphorylation of TACC3 at Ser558

**Fig. 4:**
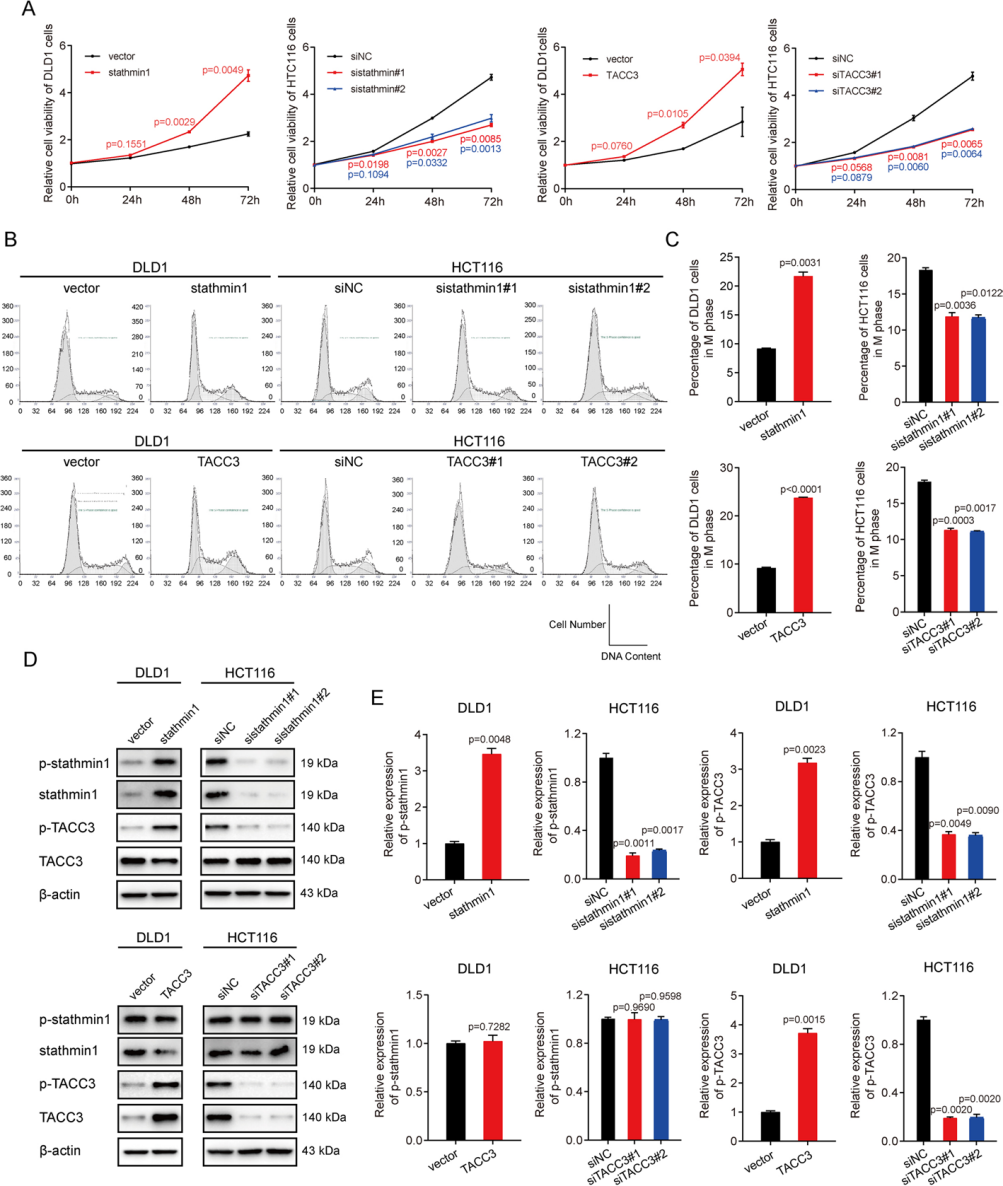
Stathmin1 positively regulates the phosphorylation of TACC3 at Ser558. **A** DLD1 cells were transfected with pCMV-stathmin1, pCMV-TACC3, or pCMV-blank vector (final vector concentrations, 2 μg/ml), and HCT116 cells were transfected with stathmin1-specific, TACC3-specific, or non-specific siRNAs (final siRNA concentrations, 20 nM). Cell viability 0, 24, 48, and 72 h after transfection was measured using MTS assays. **B** DLD1 and HTC116 cells were transfected with vectors or siRNAs as shown in **A**. Cell cycle progression was analyzed by flow cytometry 48 h after transfection. **C** Percentage of cells in **B** that were in mitotic phase. **D** DLD1 and HTC116 cells were transfected with vectors or siRNAs as shown in **A**. Samples were harvested 48 h later, and the levels of indicated factors were determined by immunoblotting. **E** Relative levels of expression of the proteins shown in **D**

The findings that stathmin1 and TACC3 are co-expressed in CRC cells because both are transcriptional products of E2F1 and that both are similarly involved in maintaining the stability of spindle fiber, suggest that these two factors may synergize with each other. To test this hypothesis, MTS cell proliferation assays were performed, in which DLD1 and HCT116 cells were transfected with vectors overexpressing stathmin1 or TACC3. Similar to findings in cells overexpressing E2F1, both stathmin1 and TACC3 were found to positively regulate cell proliferation, but the effect was not as great as that of E2F1 (Fig. 4A). Because stathmin1 and TACC3 were reported to be involved in modulating the stability of spindle fiber, we hypothesized that these factors may affect cell cycle progression in CRC cells. Flow cytometry assays of cell cycle distributions in cells overexpressing stathmin1 or TACC3 showed that both increased the proportion of mitotic cells (Fig. 4B and C).

The findings that stathmin1 and TACC3 are co-expressed and have similar functions suggest that one of these factors may regulate the functional roles of the other. Because phosphorylation of stathmin1 at Ser16 and of TACC3 at Ser558 have been reported to stabilize spindle fiber arrays, DLD1 and HCT116 cells were transfected with vectors overexpressing stathmin1 or TACC3, and the levels of expression of p-stathmin1 (Ser16) and p-TACC3 (Ser558) were determined by immunoblotting. Although enhanced expression of TACC3 had little effect on the level of phosphorylation of p-stathmin1 (Ser16), enhanced expression of stathmin1 increased the level of phosphorylation of p-TACC3 (Ser558). These results indicate that both stathmin1 and TACC3 regulated cell cycle progression, and that p-TACC3 (Ser558) was regulated by stathmin1.

### p-stathmin1 (Ser16) is essential to p-TACC3 (Ser558)

**Fig. 5:**
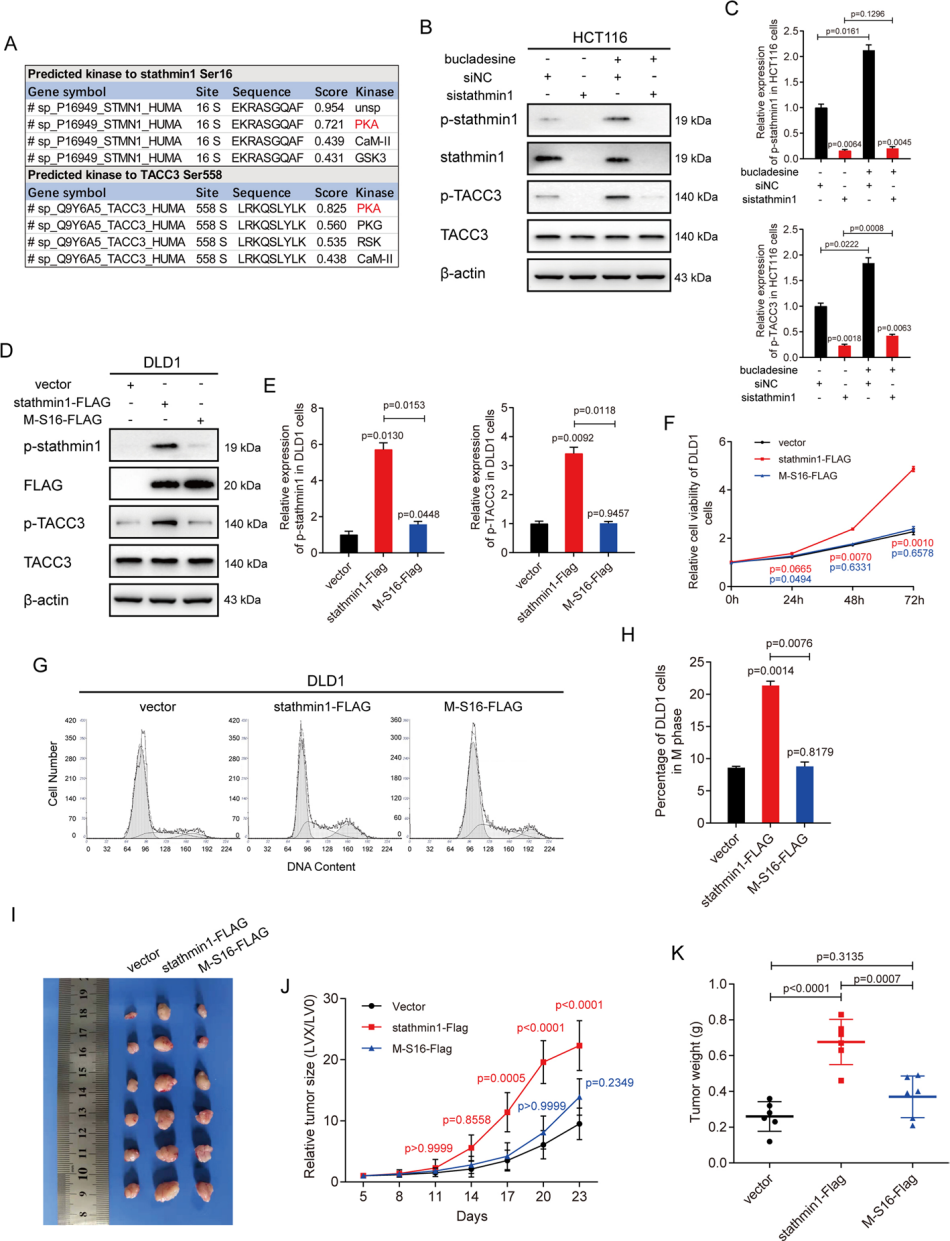
Phosphorylation of stathmin1 at Ser16 facilitates phosphorylation of TACC3 at Ser558. **A** Prediction of potential kinases targeting stathmin1 at Ser16 and TACC3 at Ser558 using the NetPhos 3.1 database. PKA was found to target both of these proteins at the indicated sites. **B** HCT116 cells were transfected with stathmin1-specific or control siRNAs (final concentrations, 20 nM). Cells were treated with 100 μM bucladesine 4 h before harvesting and harvested 48 h after transfection. The levels of indicated factors were determined by immunoblotting. **C** Relative levels of expression of the proteins indicated in **B**. **D** DLD1 cells were transfected with pCMV-stathmin-FLAG, pCMV-stathmin1-M-S16-Ala-FLAG, or pCMV-FLAG vector (final vector concentrations, 2 μg/ml) and harvested 48 h later transfection. The levels of the indicated factors were determined by immunoblotting. **E** Relative expression levels of the proteins indicated in D. **F** DLD1 cells were transfected with vectors as shown in **D**. Cell viability was measured using MTS assays 0, 24, 48, and 72 h after transfection. **G** DLD1 cells were transfected with vectors as shown in **D**. Cell cycle progression was analyzed by flow cytometry 48 h after transfection. **H** Percentage of cells in **G** in mitotic phase. **I** Representative images of tumors formed by DLD1 cells with indicated stable transfections. **J** The growth curves of the tumors formed by the indicated DLD1 cells (*N* = 6 mice per group). **K** Weight differences in tumors formed by the indicated DLD1 cells (*N* = 6 mice per group)

A search of the NetPhos 3.1 database to identify the kinase(s) targeting stathmin1 at Ser16 and TACC3 at Ser558 found both of these sites were likely phosphorylated by protein kinase A (PKA, Fig. 5A). HCT116 cells transfected with siRNA targeting stathmin1 or control siRNA were treated with bucladesine, which activates PKA, and the levels of phosphorylation of p-stathmin1 (Ser16) and p-TACC3 (Ser558) were determined. HCT116 cells transfected with control siRNA showed greater activation of both p-stathmin1 (Ser16) and p-TACC3 (Ser558) in the presence rather than in the absence of bucladesine. In contrast, cells transfected with stathmin1 siRNA showed similar levels of phosphorylation of p-TACC3 (Ser558) in the presence and absence of bucladesine, indicating that stathmin1 was essential to the phosphorylation of TACC3 at Ser558 (Fig. 5B and C).

p-stathmin1 (Ser16), along with p-TACC3 (Ser558), has been reported to stabilize microtubule arrays [12, 15]. To show that the Ser16 site was essential to the phosphorylation of TACC3 at Ser558, DLD1 cells were transfected with pCMV-stathmin1-FLAG and pCMV-stathmin1-M-S16-Ala-FLAG vectors. TACC3 phosphorylation at Ser558 was found to be activated after cells were transfected with a vector overexpressing stathmin1, but not with a vector overexpressing stathmin1 carrying a mutation at Ser16, indicating that phosphorylation of stathmin1 at Ser 16 rather than at other sites was essential to the phosphorylation of TACC3 at Ser558 (Fig. 5D and E).

Furthermore, MTS cell proliferation assay and cell cycle assessment via flow cytometry demonstrated that mutation of stathmin1 at Ser16 inhibited cell proliferation and decreased the proportion of mitotic cells (Fig. 5F–H). Xenograft tumors carrying the mutated stathmin1 grew more slowly in vivo than the tumors carrying wild type stathmin (Fig. 5I–K). Taken together, these findings indicate that p-stathmin1 (Ser16) was essential to p-TACC3 (Ser558), thereby facilitating the mitosis of CRC cells.

### p-stathmin1 (Ser16) facilitates the formation of TACC3/clathrin/α-tubulin complexes and enhances stability of spindle fiber

**Fig. 6:**
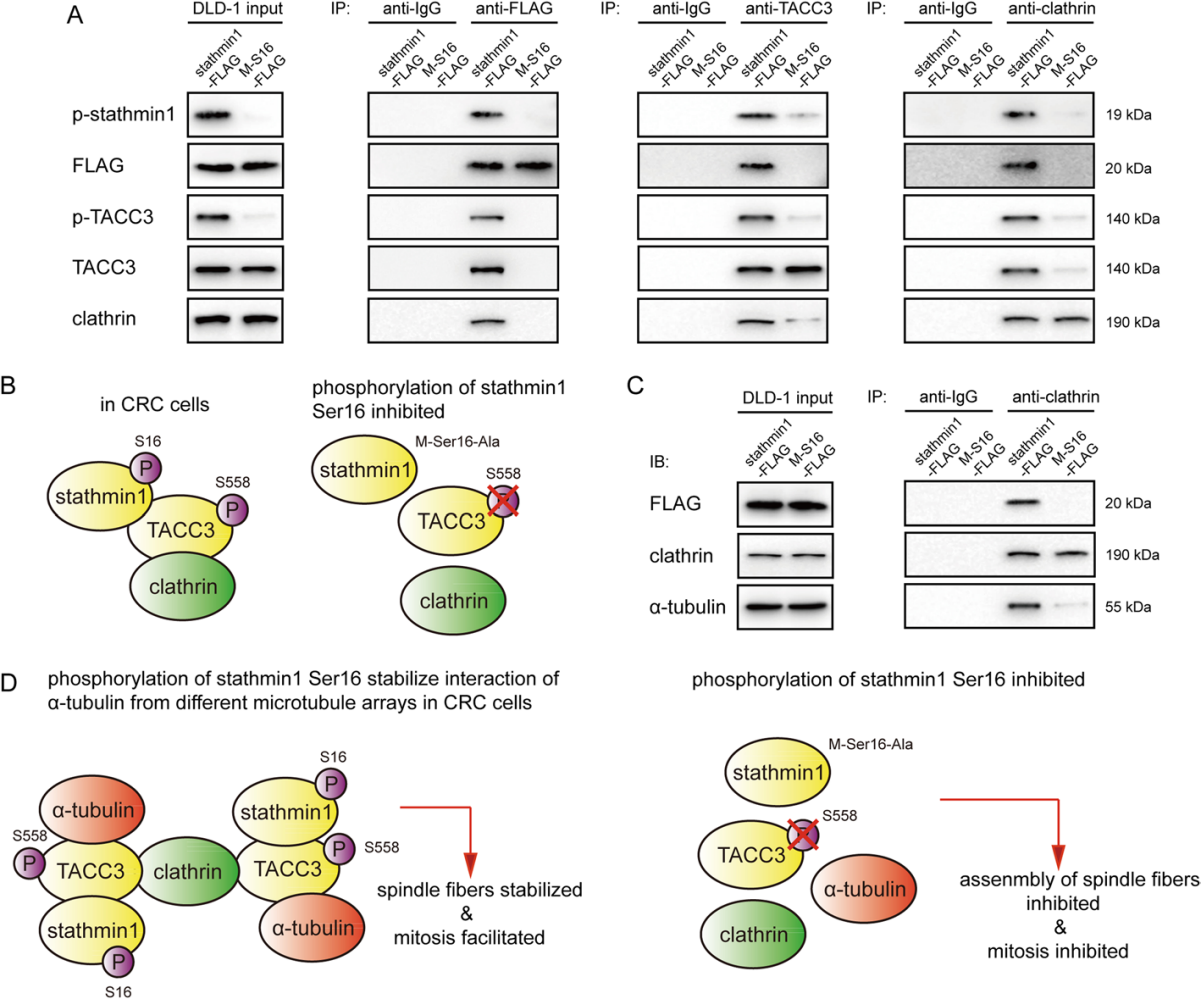
Phosphorylation of stathmin1 at Ser16 facilitates the formation of TACC3/clathrin/α-tubulin complexes and enhances the stability of spindle fiber arrays. **A** Co-immunoprecipitation and immunoblot analyses of indicated factors in DLD1 cells transfected with pCMV-stathmin-FLAG or pCMV-stathmin1-M-S16-Ala-FLAG vectors (final vector concentrations, 2 μg/ml) for 48 h. The indicated factors were immunoprecipitated using Protein A/G beads conjugated with indicated antibodies. **B** Schematic representation of the stathmin1-TACC3-clathrin interaction. Phosphorylation of stathmin1 at Ser16 facilitated its interactions with TACC3 and clathrin. **C** Co-immunoprecipitation and immunoblot analyses of indicated factors in DLD1 cells transfected with vectors as shown in A for 48 h. The indicated factors were immunoprecipitated using Protein A/G beads conjugated with indicated antibodies. **D** Schematic representation of stathmin1-TACC3-clathrin-α-tubulin interactions. Phosphorylation of stathmin1 at Ser16 facilitated the formation of TACC3/clathrin/α-tubulin complexes and enhanced the stability of spindle fiber arrays

Clathrin, the critical factor in maintaining the stability of the cytoskeleton, has been reported to interact with p-TACC3 (Ser558), but not with inactivate TACC3, to link spindle fibers [13]. Co-immunoprecipitation analyses were performed to evaluate the interaction between p-stathmin1 (Ser16) and p-TACC3 (Ser558) and to determine whether p-stathmin1 (Ser16) was essential to the interaction between p-TACC3 (Ser558) and clathrin. DLD1 cells expressing stathmin1 or the stathmin1 Ser16 mutant were lysed and treated with specific antibodies that immunoprecipitated exogenous stathmin1, endogenous TACC3, and endogenous clathrin. Co-immunoprecipitation showed that p-stathmin1 (Ser16), p-TACC3 (Ser558), and clathrin interacted with each other in DLD1 cells expressing wild-type stathmin1. However, the stathmin1 Ser16 mutant did not interact with TACC3, reducing the level of phosphorylation of p-TACC3 (Ser558) and inhibiting the interaction of p-TACC3 (Ser558) with clathrin (Fig. 6A and B).

**Fig. 7:**
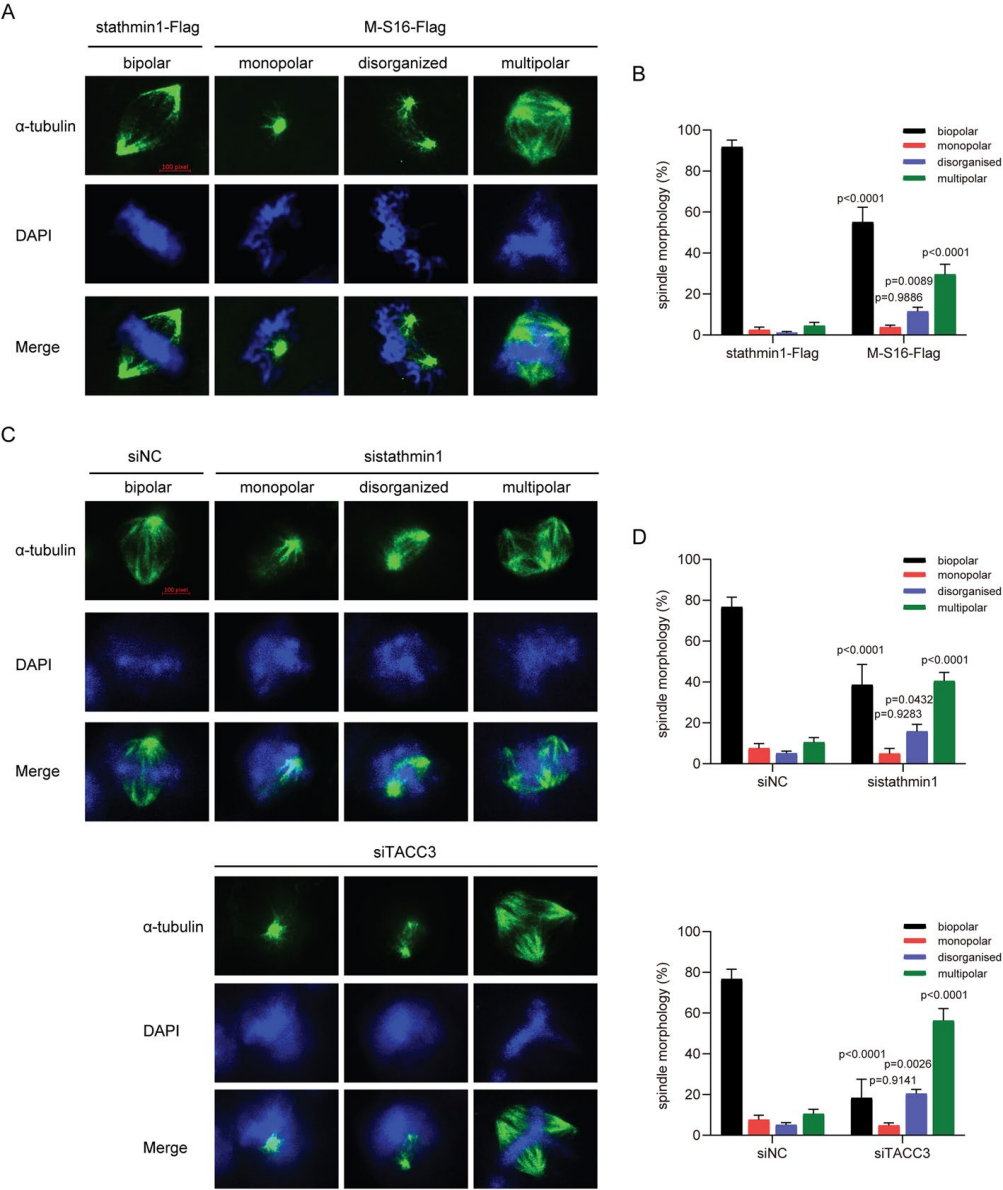
Stathmin1 mutation at Ser16 or depletion of stathmin1 or TACC3 induces the formation of aberrant spindles. **A** Induction of multipolar and disorganized spindles in DLD1 cells transfected with a plasmid expressing a Ser16-mutated or wild-type stathmin1. After transfection for 48 h, the cells were fixed and stained with antibody to α-tubulin and DAPI. Bar, 10 pixels. **B** Quantification of the numbers of aberrant spindles in the DLD1 cells described in **A**. One hundred cells in each group were counted. **C** Induction of multipolar and disorganized spindles in HCT116 cells transfected with stathmin1, TACC3, or non-specific siRNA. After transfection for 48 h, cells were fixed and stained with antibody to α-tubulin antibody and DAPI. Bar, 10 pixels. **D** Quantification of the number of aberrant spindles in the HCT116 cells described in **C**. One hundred cells in each group were counted

Phosphorylated TACC3 (Ser558) has been shown to interact with microtubule-related molecules, with clathrin linking p-TACC3 (Ser558) on different spindle fibers to stabilize spindle fiber. To evaluate the effect of p-stathmin1 (Ser16) on the stability of spindle fiber, clathrin was co-immunoprecipitated with its interacting proteins in cells overexpressing stathmin1 or the stathmin1 Ser16 mutant. Clathrin was found to strongly interact with α-tubulin in cells overexpressing wild-type, but not Ser 16 mutant, stathmin1 (Fig. 6C). Because clathrin was regarded as a bridge linking different spindle fibers, it was found to indirectly interact with α-tubulin on different spindle fiber by binding to p-TACC3 (Ser558). The absence of p-stathmin1 (Ser16) inhibited the p-TACC3 (Ser558) and the link between p-TACC3 (Ser558) and clathrin1, thereby suppressing the formation of TACC3/clathrin/α-tubulin complexes and reducing the stability of spindle fiber arrays (Fig. 6D). The effect of the stathmin1 mutation on spindle assembly in CRC cells was therefore examined by immunofluorescence analysis, which showed that the Ser16-mutated stathmin1 induced spindle aberrations, such as disorganized and multipolar spindles, in DLD1 cells (Fig. 7A and B). Knockdown of stathmin1 or TACC3 also caused spindle aberrations in HCT116 cells (Fig. 7C and D).

### E2F1-induced stathmin1 and TACC3 are associated with poor prognosis in CRC patients

**Fig. 8:**
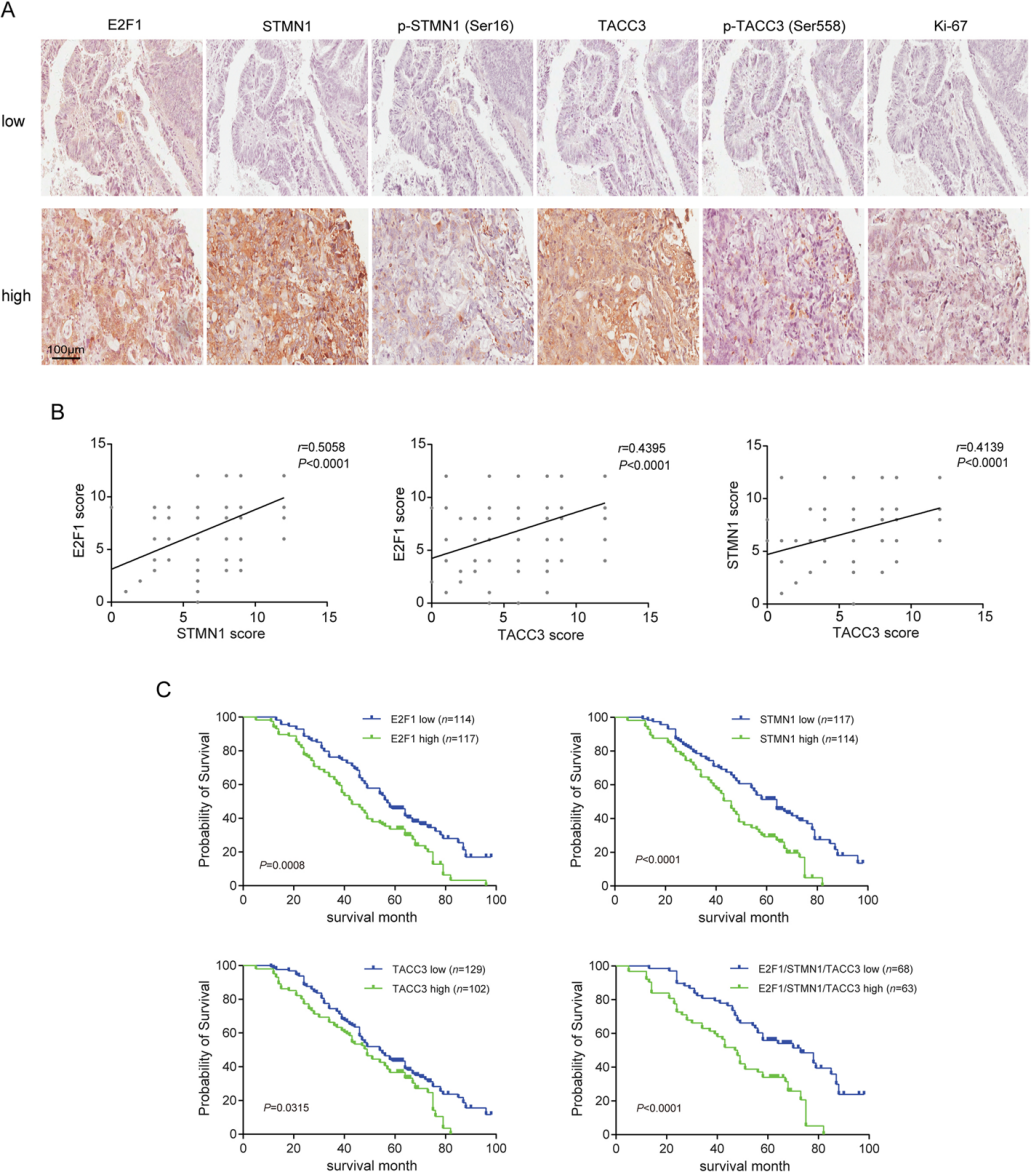
E2F1-induced stathmin1 and TACC3 are associated with a poor prognosis in CRC patients. **A** Immunohistochemical analysis of the levels of E2F1, stathmin1, p-stathmin1, TACC3, p-TACC3, and Ki-67 in pathological CRC tissues (scale bar, 100 μm). Representative images of two CRC patients are shown. **B** Correlation among concurrent immunostaining scores of E2F1, stathmin1, and TACC3 in CRC tissues. Spearman’s test was used to analyze the correlations. **C** Overall survival of probability of CRC patients with high (*n* = 117) and low (*n* = 114) expression of E2F1, high (*n* = 114) and low (*n* = 117) expression of stathmin 1, high (*n* = 102) and low (*n* = 129) expression of TACC3, and high (*n* = 63) and low (*n* = 68) expression of E2F1/stathmin1/TACC3

To assess the roles of E2F1-induced stathmin1 and TACC3 in colon tumorigenesis, tumor specimens were collected from 231 patients with CRC. Immunohistochemical analyses showed that samples with high levels of E2F1 expression also had higher levels of stathmin1, p-stathmin1, TACC3, p-TACC3, and KI67 than those with lower E2F1 expression (Fig. 8A). Further analyses indicated positive correlations among the levels of E2F1, TACC3, and stathmin1 in CRC tissues (Fig. 8B). Analysis of clinical relevance showed that higher levels of expression of E2F1, stathmin1, and TACC3 were positively associated with tumor size (E2F1 may relate to tumor size, although the *P*-value was > 0.05), lymph node metastasis, TNM stage, and distant metastasis, but not with other clinicopathological characteristics (Table 1). Besides, age, differentiation, and distant metastasis were the risk factors for overall survival in multivariate analysis (Table 2). Kaplan–Meier analysis showed that overall survival rates were poorer in patients with high than low levels of E2F1, stathmin1, and TACC3 (Fig. 8C and Table 2). These results indicate that E2F1 is positively correlated with stathmin1 and TACC3 in tumor tissues derived from CRC patients, and that these proteins are involved in CRC development.

**Table 1:** Association of the expression of E2F1, stathmin1, and TACC3 with clinicopathological features in CRC

	Cases	E2F1 expression			STMN1 expression			TACC3 expression		
		Low	High	*P*-value	Low	High	*P*-value	Low	High	*P*-value
		Cases	Cases		Cases	Cases		Cases	Cases	
	231	114	117		117	114		129	102	
Tumor location										
Colon	97	46	51	0.8596	51	46	0.1869	55	42	0.7685
Rectum	134	68	66		66	68		74	60	
Gender										
Male	124	55	69	0.2213	65	59	0.4271	66	58	0.0915
Female	107	59	48		52	55		63	44	
Age										
≤ 65	93	42	51	0.5457	48	45	0.9476	52	41	0.9877
> 65	138	72	66		69	69		77	61	
Differentiation status										
Well	50	23	27	0.1206	27	23	0.7617	28	22	0.7723
Moderate	154	84	70		79	75		88	66	
Poor	27	7	20		11	16		13	14	
Tumor size										
< 5 cm	110	56	54	0.0729	63	47	0.0208*	70	40	0.0247*
≥ 5 cm	121	58	63		54	67		59	62	
LNM										
N0	130	72	58	0.0214*	76	54	0.0040*	79	51	0.0108*
N1	73	34	39		32	41		37	36	
N2	28	8	20		9	19		13	15	
TNM										
I	35	19	16	0.0030*	21	14	0.0014*	16	19	0.0001*
II	88	46	42		47	41		58	30	
III	89	45	44		46	43		51	38	
IV	19	4	15		3	16		4	15	
Distant metastasis										
M0	212	110	102	0.0002*	114	98	0.0001*	125	87	< 0.0001*
M1	19	4	15		3	16		4	15

**Table 2:** Multivariate and univariate analysis of prognostic factors for OS

Variable	Multivariate			Univariate		
	HR	95% CI	*P*-value	HR	95% CI	*P*-value
Tumor location (colon versus rectum)	0.984	0.725–1.333	0.9130	1.058	0.770–1.453	0.7280
Gender (male versus female)	0.875	0.646–1.174	0.3728	0.960	0.701–1.314	0.7987
Age (≤ 65 years versus > 65 years)	1.439	1.086–1.973	0.0154	1.374	0.981–1.923	0.0642
Tumor size (< 5.0 cm versus ≥ 5.0 cm)	1.238	0.926–1.680	0.1540	1.108	0.809–1.517	0.5231
LNM (N0 versus N1/2)	0.999	0.740–1.348	0.9929	0.847	0.606–1.185	0.3331
Differentiation (well and moderate versus poorly)	1.667	1.157–3.184	0.0133	1.333	0.833–2.134	0.2306
Distant metastasis (no versus yes)	2.046	1.419–5.531	0.0035	1.737	1.008–2.981	0.0466
E2F1 expression (low versus high)	1.642	1.255–2.290	0.0008	1.332	0.892–1.989	0.1608
STMN1 expression (low versus high)	1.877	1.496–2.754	< 0.0001	1.707	1.165–2.502	0.0061
TACC3 expression (low versus high)	1.373	1.041–1.924	0.0315	0.899	0.620–1.302	0.5727

## Discussion

**Fig. 9:**
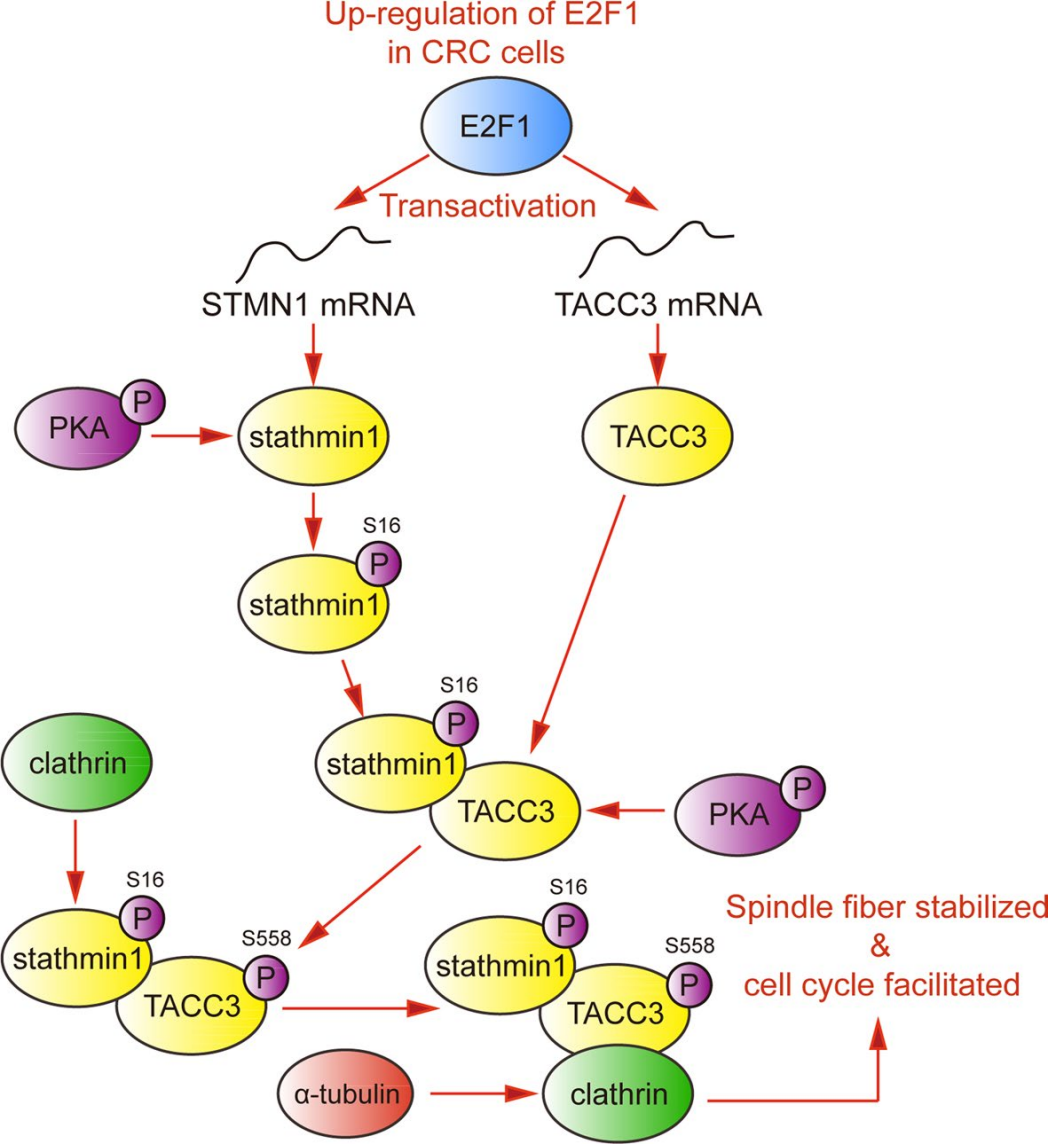
Overview of the E2F1-stathmin1-TACC3 axis in CRC cells. E2F1 transactivated stathmin1 and TACC3, and stathmin1 phosphorylated at Ser16 facilitated the formation of the TACC3/clathrin/α-tubulin complex, which promoted cell cycle progression by enhancing the stability of spindle fiber arrays in CRC cells

In this study, we found that E2F1 transactivated stathmin1 and TACC3, two factors that stabilize spindle fibers, thus promoting the cell cycle progression of CRC (Fig. 9). Clinical analyses showed that E2F1 correlated positively with stathmin1 and TACC3 in CRC tissues, and their high expressions predicted poor survival in CRC patients.

Rapid proliferation and liver metastases of CRC are the main causes of CRC-related deaths. Oxygen supply is sufficient in colorectal and CRC tissues because of their large surface areas of colorectal epithelial cells and the high number of blood vessels supplying these tissues. This provides an excellent microenvironment for tumor proliferation, making it necessary to identify strategies that inhibit CRC proliferation [20]. Although many factors have been reported to contribute to CRC proliferation, the mechanism remains poorly understood.

E2F1 is a critical transcription factor highly expressed in most cancer tissues [21]. E2F1 has been shown to transactivate CDKs and facilitate cell proliferation by accelerating cell cycle progression. Although E2F1 has been widely reported to promote cell cycle progression through the CDK3-pRB-E2F1 axis, the downstream genes regulated by E2F1 are multi-functional in the cell cycle [5]. In detail, E2F1 interacts with DP1 or DP2 to promote cell cycle progression by transactivating thymidylate synthase, cyclin A, cyclin E, and cdk2. In our previous work, increased E2F1 was required for the proliferation of CRC cells by inducing the expression of ribonucleotide reductase M2, which provided dNTPs for DNA synthesis [22]. Besides, spindle organization is also essential during mitosis; however, the involvement of E2F1 in this biological process is unclear. Here, we screened and analyzed 51 genes related to spindle organization in the E2F1-regulated gene subset, in which stathmin1 and TACC3, two microtubule-binding factors, were strictly regulated by E2F1. Given that TACC3 and stathmin1 stabilize spindle fibers, E2F1 was thought to facilitate cell cycle progression of CRC by inducing stathmin1 and TACC3. Considering the similar DNA element recognized by E2Fs, other E2F members may also affect the expressions of stathmin1 and TACC3 to some extent. Because different E2F members have diverse transcriptional properties and different cancer types have distinct expression profiles of E2Fs, it is worth investigating E2Fs-regulated spindle organization in the context of other cancers.

Stathmin1 has been found to be a cytosolic phosphoprotein that regulates microtubule dynamics during mitosis, and is associated with aggressive cellular behaviors, especially proliferation, in a wide spectrum of human malignancies [23–25]. Abnormal expression of stathmin1 was validated in CRC tissues, and CRC patients with higher expression of stathmin1 have poorer prognosis [26]. Reportedly, stathmin1 levels are upregulated when retinoblastoma protein (RB) is inactivated and E2F is deregulated [27]. Notably, our findings validated that stathmin1 was transactivated by E2F1 in CRC cells. Physiologically, unphosphorylated stathmin1 causes microtubule depolymerization by sequestering soluble tubulin, and induces microtubule catastrophe [28]. Four serine phosphorylation sites (Ser16, Ser25, Ser38, and Ser63) were identified at the stathmin1 protein, and their phosphorylation affected the microtubule destabilizing ability of stathmin1 [12, 29]. In early mitosis, phosphorylation of stathmin1 overrides its microtubule destabilizing ability, allowing the formation of mitotic spindles, while stathmin1 is dephosphorylated when cells exit mitosis and undergo cytokinesis [30]. In the above studies, cyclin-dependent protein kinases (CDKs) confer the phosphorylation of Ser25 and Ser38, and p21-activated kinase (Pak) directly phosphorylates Ser16 [12, 29]. However, we predicted Ser16 as a target of PKA and found that dibutyryl-cAMP (Bucladesine, a PKA activator) induced the phosphorylation of Ser16, suggesting that PKA may serve as a novel kinase for stathmin1 phosphorylation (Ser16). As reported by Larsson et al., although dual phosphorylation on Ser25 and Ser38 appears to be required for phosphorylation of Ser16, by themselves, Ser25 and Ser38 are of only minor importance in direct regulation of stathmin1 activity. Subsequent phosphorylation of Ser16 may switch off its activity [12]. Consistently, we found that p-stathmin1 (Ser16) was required for TACC3 phosphorylation and further mitotic spindle assembly.

As a member of the TACC family characterized by a highly conserved C-terminal coiled-coil domain, TACC3 functions as a microtubule stabilizer in the process of mammalian mitotic spindle assembly, thereby regulating centrosome and microtubule dynamics [13, 31]. TACC3 is highly expressed in a variety of tumors including colorectal cancer, and promotes tumor progression partially by increasing cell proliferation [10, 32]. TACC3 deletion was found to suppress the growth of intestinal tumors in vitro and in vivo by perturbing the mitotic spindle of intestinal stem cells [33]. Clinical investigations indicated TACC3 expression as an independent prognostic factor for CRC patients, which correlated with poor survival [32]. Mechanistically, localization of TACC3 to mitotic spindles is mediated by phosphorylation on Ser558, which could be induced by the mitotic kinase Aurora A in CRC cells [34]. Besides, we predicted PKA as a potential kinase targeting Ser558, which is worth further verification. Interestingly, the present study showed that p-stathmin1 (Ser16) was also indispensable for the activation of p-TACC3 (Ser558), with the latter facilitating the formation of TACC3/clathrin/α-tubulin complexes and stabilizing mitotic spindles. Because stathmin1 and TACC3 were co-regulated by E2F1 in CRC cells, TACC3 might exert a synergistic effect with stathmin1 during mitosis.

## Conclusions

Functionally, activated stathmin1 and TACC3 are thought to stabilize spindle fibers, but no study to date had shown a functional link between stathmin1 and TACC3. Our study found that E2F1 induced the expressions of both stathmin1 and TACC3, while phosphorylation of TACC3 at Ser558 was dependent on p-stathmin1 (Ser16), consequently facilitating the mitosis and proliferation of CRC cells.

## Supplementary Information

**Additional file 1: Table S1.** Sequences of siRNAs. **Table S2.** Primers for ChIP analysis.

## Data Availability

The datasets used and/or analysed during the current study are available from the corresponding author on reasonable request.

## Abbreviations

TACC3: Transforming acidic coiled-coil-containing protein 3
CRC: Colorectal cancer
PKA: Protein kinase A
qPCR: Quantitative PCR
SDS-PAGE: Sodium dodecyl sulfate–polyacrylamide gel electrophoresis
ChIP: Chromatin immunoprecipitation
ANOVA: Analysis of variance
TCGA: The Cancer Genome Atlas
KEGG: Kyoto Encyclopedia of Genes and Genomes
