# Supplementary material for: E2F1 promotes cell cycle progression by stabilizing spindle fiber in colorectal cancer cells
Source: Cell Mol Biol Lett. 2022 Oct 11;27:90. doi: 10.1186/s11658-022-00392-y (PMC9552509; doi:10.1186/s11658-022-00392-y)
Supplement: Supplementary file 1 — Additional file 1: Table S1. Sequences of siRNAs. Table S2. Primers for ChIP analysis. [file 11658_2022_392_MOESM1_ESM.docx]

**Table S1. Sequences of siRNAs**

| Targets | Sense and antisense chains (5’-3’) |
| --- | --- |
| siE2F1#1 | S: CGCUAUGAGACCUCACUGATT |
|  | A: UCAGUGAGGUCUCAUAGCGTT |
| siE2F1#2 | S: GACAUCACCAACGUCCUUGTT |
|  | A: CAAGGACGUUGGUGAUGUCTT |
| sistathmin1#1 | S: AGCACGAGAAAGAAGUGCUTT |
|  | A: AGCACUUCUUUCUCGUGCUTT |
| sistathmin1#2 | S: UGGAGGAAAUUCAGAAGAATT |
|  | A: UUCUUCUGAAUUUCCUCCATT |
| siTACC3#1 | S: CAGGAAGUUCUGAGAACCATT |
|  | A: UGGUUCUCAGAACUUCCUGTT |
| siTACC3#2 | S: CAGUCCUUAUACCUCAAGUTT |
|  | A: ACUUGAGGUAUAAGGACUGTT |
| siNC | S: GCGACGAUCUGCCUAAGAUTT |
|  | A: AUCUUAGGCAGAUCGUCGCTT |

*Sequences of siRNAs were based on shRNA sequences targeting specific target shown on the website of Sigma- Aldrich.

**Table S2. Primers for ChIP analysis**

| Primers | Sequences (5’-3’) |
| --- | --- |
| *STMN1* promoter (-1340/-1330) | F: GAAGCCAAGTGGCATTGCTAA |
|  | R: TGCCACACAAAAGTCTCACATTC |
| *STMN1* promoter (-879/-869) | F: GGTCCTGCTCACAGGCTAAG |
|  | R: ACAACTAAGGGGTGCCCAAG |
| *STMN1* promoter (-139/-132) | F: TTCCTCCCCTATCATCCGCA |
|  | R: CTCACTGTGTGCGTTCTTGC |
| *TACC3*promoter (-771/-761) | F: CAAGTGGAAGGGCACGAAG |
|  | R: TCTCGCCTATCTGGTGTTCG |
| *TACC3*promoter (-160/-150) | F: CTCCACTCCGGAAACGGAAC |
|  | R: TTCGCGCGGCCAATCA |
| *TACC3*promoter (+91/+101) | F: GGCCATCAAGGGCTAGAAGC |
|  | R: TGTCGTTTAAGACCTGCAGAC |
